# Visual Plasticity in Adults

**DOI:** 10.1155/2017/8469580

**Published:** 2017-06-04

**Authors:** Jiawei Zhou, Zili Liu, Simon Clavagnier, Alexandre Reynaud, Fang Hou

**Affiliations:** ^1^School of Ophthalmology and Optometry and Eye Hospital, Wenzhou Medical University, Wenzhou, Zhejiang, China; ^2^Department of Psychology, University of California Los Angeles, Los Angeles, CA, USA; ^3^McGill Vision Research, Department of Ophthalmology, McGill University, Montreal, QC, Canada

The pioneering work of Hubel and Wiesel established the concept of a critical period for visual development early in life. During this period, cortical development is most susceptible to the alteration of sensory experience by the environment or other factors. Although this is a well-accepted concept in neurobiology, it is also now recognized that there is some limited plasticity remains after a critical period well into adulthood. A range of visual functions in adult subjects can be modified as a result of perceptual learning, brain stimulation, or adaptation, some with effects that can last very long, up to several months [1].

Perceptual learning can be achieved through intensive training during which a subject repeats a perceptual detection or discrimination task over and over again [2]. Noninvasive brain stimulation techniques such as transcranial magnetic stimulation (TMS) and transcranial direct current stimulation (tDCS) have also been shown to induce functional change in the adult brain [3, 4]. Finally, plastic changes can also be induced by visual adaptation and deprivation [5, 6]. These procedures seem very promising in the perspective of developing new clinical therapies, such as the treatment of adult amblyopia [7]. Hence, this special issue aims to provide a kaleidoscope that presents novel findings in adult visual plasticity from whole brain network to human behavior.

Two studies tackled directly with clinical cases. The first one focused on the plastic changes occurring in congenital and late blindness. With tractography based on diffusion-weighted magnetic resonance imaging and diffusion tensor imaging, N. H. Reislev et al. observed plastic microstructural changes in thalamocortical connectivity of blind people that could not be captured at the network level. The other one assessed the sensory eye dominance in treated anisometropic amblyopes. Y. Chen et al. showed that the treated anisometropic amblyopes, even those with a normal range of visual acuity, exhibited abnormal binocular processing. Their results thus suggest that there is potential for improvement in treated anisometropic amblyopes that may further enhance their binocular visual functioning.

Then, two other studies examined how short-term changes can be driven by exogenous stimuli. Y. Chen et al. tested the effect of different types of ambient illumination during 3D TV viewing on ocular status. They observed that, in general, 3D TV viewing modifies the ocular status of adults. A front illumination has less impact on the accommodative response and microfluctuation, suggesting that this type of illumination is the most appropriate mode for 3D TV viewing. On the other hand, M.A. De Niear et al. investigated the impact of feedback in multisensory temporal recalibration. They concluded that feedback signals promote and sustain audiovisual recalibration over the course of cumulative learning and enhance rapid trial-to-trial learning.

Lastly, it has recently been shown by Lunghi et al. [6] that short-term monocular deprivation alters binocular balance. In this special issue, P. Binda and C. Lunghi extended their previous work and illustrated how plastic effects due to monocular deprivation can be assessed physiologically. They demonstrated that slow pupil oscillation amplitude increased after monocular deprivation, with larger changes correlated with larger ocular dominance changes measured by binocular rivalry. Their findings suggest that there might be a common mechanism shared by the slow pupil oscillation and ocular balance. Instead of using binocular rivalry as used in the other study [8], J. Zhou et al. investigated the effect of aerobic exercise on the monocular deprivation effect using a binocular fusion task. They found no additional effect of exercise after short-term monocular occlusion and argued that the enhancement of ocular dominance plasticity from exercise could not be generalized to all visual functions.

Altogether, these studies will contribute to a better understanding of how plastic changes can occur or can be induced in our visual system. These could potentially contribute to the new clinical therapies for developmental visual disorders such as amblyopia.
